# Yohimbine-Induced Reactivity of Heart Rate Variability in Unmedicated Depressed Patients With and Without Adverse Childhood Experience

**DOI:** 10.3389/fpsyt.2021.734904

**Published:** 2021-12-16

**Authors:** Christian Eric Deuter, Christian Otte, Katja Wingenfeld, Linn Kristina Kuehl

**Affiliations:** ^1^Charité Universitätsmedizin Berlin, Corporate Member of Freie Universität Berlin, Humboldt-Universität zu Berlin, and Berlin Institute of Health, Campus Benjamin Franklin, Department of Psychiatry and Psychotherapy, Berlin, Germany; ^2^Department of Psychology, Clinical Psychology and Psychotherapy, MSB Medical School Berlin, Berlin, Germany

**Keywords:** childhood trauma, early life maltreatment, adverse childhood experience, heart rate variability, major depressive disorder, yohimbine, norepinephrine, parasympathetic and sympathetic reactivity

## Abstract

Stressful life events play a role in the pathogenesis of major depressive disorder (MDD) and many patients with MDD were exposed to developmental stress due to adverse childhood experiences (ACE). Furthermore, dysregulation of the autonomic nervous system and higher incidence of cardiovascular disease are found in MDD. In MDD, and independently in individuals with ACE, abnormalities in heart rate variability (HRV) have been reported. While these are often confounded, we systematically investigated them with a study which included MDD patients with/without ACE as well as healthy individuals with/without ACE. With this study, we investigated the influence of noradrenergic stimulation on HRV reactivity in unmedicated participants in a randomized, double-blind, repeated measures design. Our sample consisted of men and women with MDD and ACE (*n* = 25), MDD without ACE (*n* = 24), healthy participants with ACE (*n* = 27), and without ACE (*n* = 48). Participants received a 10 mg single dose of the alpha-2 antagonist yohimbine that increases noradrenergic activity or placebo on 2 separate days, with ECG recordings before and after drug administration at defined intervals. We found lower basal HRV in MDD and ACE: patients with MDD had reduced RMSSD whereas participants with ACE had lower LF-HRV. Contrary to our hypothesis, there was no effect of yohimbine. With this study, we were able to replicate previous findings on HRV differences in MDD and ACE. From the null effect of yohimbine, we conclude that the yohimbine-induced sympathetic activation is not a significant driver of HRV in MDD and ACE.

## Introduction

Major Depressive Disorder (MDD) is associated with a dysregulation of the autonomic nervous system (ANS), reflected in a disturbed balance between the sympathetic and parasympathetic branch of the ANS (1–3). Among several other adverse health effects, MDD increases the risk of developing cardiovascular disease and ultimately of cardiac mortality (4). Numerous studies have demonstrated that patients with MDD, and independent of comorbid cardiovascular disease, have lower heart rate variability (HRV) when compared to healthy controls, both under resting condition and measured as reactivity after stress (5, 6). Heart rate variability describes the natural variation of the time interval between successive heartbeats and reflects the ability of the organism to constantly adapt the heart rate to current demands. Acute stress, physical exercise, or mental load require an increase in heart rate, which normally decreases again when the body is relieved of workload and relaxed. Lower variability of the heart rate can therefore be interpreted as an reduced adaptability of the organism, i.e., lower capacity to adjust to current demands (7). The organism adjusts the heart rate *via* autonomous physiological regulatory pathways. While it is established that high frequency changes in heart rate are mediated by the vagus nerve and therefore the parasympathetic nervous system, the hypo-reactivity found in MDD patients could as well be attributed to constant sympathetic dysregulation (8–10).

Along with adrenaline, noradrenaline (NA) is the main transmitter of the sympathetic nervous system and is also involved in numerous cognitive processes in the brain (11). Noradrenaline metabolism and the transmission processes based on this neurotransmitter are also disturbed in MDD (12). This involves both the concentration levels measured in patients' blood and receptor sensitivity, whereby the findings are inconsistent to some degree and numerous questions remain to be elucidated (13). In depressed patients, lower NA levels are found and antidepressant medication that increases the availability of NA is therapeutically effective (14, 15). Yet, after stress, the release of NA is extensively increased–and chronic stress is a major risk factor for the development of MDD, patients often report increased stress leading up to a depressive episode, an alleged contradiction which has even been termed “the noradrenergic paradox” (16). However, this paradox might be resolved by taking receptor level alterations into account. For instance, a higher density and sensitivity of alpha2- adrenergic receptor in the brains of MDD patients have been reported (17, 18). The alpha2-receptor essentially controls the autoregulation of NA release in the central nervous system, primarily *via* the Locus Coeruleus (LC) region in the brainstem. The increased release of NA after stress, in addition to an increased affinity of NA-binding auto-receptors in the LC, could lead to a subsequent reduction of transmitter levels and compensatory changes in the LC in MDD patients (12, 16).

Among patients with MDD, difficult, often traumatizing, experiences in childhood, and adolescence are frequently found, which are commonly referred to as adverse childhood experiences (ACE). These experiences are associated with chronic stress in important developmental stages and have a lasting influence on psychological and physiological processes in adulthood, potentially *via* epigenetic mechanisms (19). Different studies have shown increased stress reactivity of the sympathetic nervous system in adult subjects with traumatic experiences in childhood, accompanied by increased NA release (20, 21). Similar to MDD, increased alpha2 receptor sensitivity was reported in individuals with ACE (22). The autonomic dysregulation described above should be most pronounced in the subpopulation of MDD patients whose biographies are influenced by ACE such as physical or sexual abuse.

Taken together, these findings indicate that persistent autonomic dysregulation is present in both patients with MDD and individuals with ACE, which may represent a causal mechanism for lower HRV. However, the two factors usually do not occur independently of each other: ACE represents one of the most important influences in the pathogenesis of MDD, and numerous patients report previous ACE (23). As with MDD, individuals with ACE show lower resting HRV (24), and also blunted HRV reactivity to stress (25, 26). The joint presence of both factors leads to a compounding effect: some studies have demonstrated the strongest HRV abnormalities in patients with MDD and early traumatic experiences, compared with MDD patients without them or healthy controls (27). Another study that examined the joint influence of MDD and ACE on stress-related HRV reactivity found that MDD patients with a trauma history exhibited the lowest HRV after stress exposure (28).

In this regard, numerous of the aforementioned studies have exclusively examined HRV parameters that reflect the activity of the parasympathetic branch of the ANS. Although this seems justified by the fact that the vagal control of these parameters is well-established, this represents only a part of the autonomic cascade. It could be shown that especially sympathetic, and not parasympathetic activation, mediates stress-related HRV reactivity in individuals with ACE (29), and sympathetic over-activation, alongside with vagal withdrawal, has been proposed as an explanatory mechanism for altered HRV stress reactivity in MDD (6).

With this study, we systematically investigated both MDD and ACE in a full factorial, fully crossed orthogonal design, which included MDD patients with/without ACE as well as healthy individuals with/without ACE. We specifically aimed to investigate the influence of sympathetic autonomic activation on HRV reactivity in these groups. For this purpose, the alpha2-adrenergic antagonist yohimbine was administered and ECG was recorded over 5 min periods once before and three times after administration at fixed intervals. Because our manipulation was targeted at direct and specific sympathetic activation, low frequency (LF)-HRV, that primarily reflects variations in cardiac sympathetic activity, served as our primary outcome measure (5). Since a complex interplay between the sympathetic and parasympathetic nervous systems can be assumed, we also report those well-established parameters that are primarily associated with the parasympathetic nervous system, i.e., the root mean square of successive differences (RMSSD) in the time domain and the high frequency (HF)-HRV for the frequency domain. RMSSD and HF-HRV reflect cardiac vagal activity and, in addition, RMSSD is comparatively free of respiratory influences (8). With the selection of these outcome measures, we also ensure comparability to previous studies in the same research area, which are predominantly based on these 3 parameters (5, 6, 24, 30).

Based on previous literature, we expected lower basal HRV across all parameters in patients with MDD and individuals with ACE. Both factors should interact, so that the lowest values should be found in depressed patients with a history of ACE. However, we were agnostic about the relative contribution of the two group factors. After administration of yohimbine, we expected an increase in LF-HRV and a decrease in the vagally-mediated parameters RMSSD and HF-HRV across groups. This reduction should have been more pronounced in MDD and ACE due to the higher alpha2-adrenoceptor sensitivity. Again, we had expected a summative effect of both factors.

## Methods

### Participants

We recruited patients with MDD and healthy participants through public announcements and from our special department for affective disorders at the Department of Psychiatry and Psychotherapy, Campus Benjamin Franklin, Charité Universitätsmedizin Berlin. Healthy participants and outpatients received monetary compensation (100 €) for their participation. The study was approved by the ethics committee of the Deutsche Gesellschaft für Psychologie (DGPs, German Society of Psychology) and carried out in accordance with the Declaration of Helsinki, all participants gave their written informed consent.

Patients with MDD were included if they met criteria for a current MDD episode as assessed by a trained psychologist using a German version of the Structured Clinical Interview for DSM-IV Axis I (SCID-I) to validate psychiatric diagnoses (31). In addition to the SCID-I interview, current depressive symptoms were assessed by a clinical rating scale and questionnaire, the Montgomery Asberg Depression Rating Scale (MADRS) (32, 33), and the Beck Depression Inventory (BDI) (34). Adverse childhood experiences were defined as repeated physical or sexual abuse at least once a month over duration of a year or more. These had to occur during childhood or adolescence, i.e., before age 18. Again, criteria were assessed by a semi-structured interview, the validated German version of the Early Trauma Inventory (35, 36). In addition, ACE was assessed with a German version of the Childhood Trauma Questionnaire (CTQ) (37, 38). In the MDD groups, schizophrenia, schizoaffective disorder, bipolar disorder, depressive disorder with psychotic features, dementia, alcohol or drug abuse, and panic disorder led to exclusion. Healthy participants had no current diagnosis of a mental disorder and no previous diagnosis of MDD. Additional exclusion criteria for all participants included CNS-related disorders, neurological disorders, severe somatic illness, type 1 and 2 diabetes, steroid disorders, hypertension, current infections, pregnancy, and use of psychotropic medications. Physical health criteria were assessed by physical examination, clinical interview, and blood test.

Of the participants who completed the measurement on both days, data from 12 participants were incomplete due to a technical or recording malfunction. Two participants were excluded from the analysis because the values in our dependent measures deviated abnormally (for this purpose, the average per participant per session was regarded; outliers were defined as deviation by more than three standard deviations from the mean of the total sample in at least two of the three parameters in both sessions). The final data set consisted of 124 participants: 25 MDD patients with ACE (MDD+/ACE+), 24 MDD patients without ACE (MDD+/ACE−), 27 participants with ACE but without current or lifetime MDD (MDD−/ACE+), and 48 participants without current or lifetime MDD and no ACE (MDD−/ACE−).

### Procedure

Participants came for 3 separate sessions. During the first appointment, participants underwent a physical examination and blood sampling. Afterwards, diagnostics and assessment of MDD and ACE with questionnaires and structured interviews took place. Experimental testing was conducted on 2 separate days. On 1 day, the participants received 10 mg of oral yohimbine (Spiegel, DESMA), on the other day a placebo (p-Tabletten, Lichtenstein). Experimental order was counterbalanced across participants; drug administration was double blinded to participant and experimenter.

Experimental testing started at 9:30 h in the morning. Participants had to refrain from physical activity and caffeine consumption on these mornings and from eating, drinking and smoking 30 min prior to the start of the experiment. Participants were seated upright in a comfortable chair. Drug administration took place at 9:45, and the entire study lasted until 12:15. Participants were at rest, filled out questionnaires and were allowed to read for 1 h after drug administration. Multiple experimental psychological tests were part of the study, the results of which have been reported elsewhere (34, 39, 40). We conducted ECG recordings at four different times, for 5 min each: at 9:40, immediately before drug administration (baseline), at 10:35, 11:15, and 11:45.

### Assessment of Heart Rate Variability

We recorded electrocardiogram (ECG) using Tyco Healthcare H34SG Ag/AgCl ECG electrodes and a Biopac MP150 amplifier system at 1 kHz sampling rate, with a hardware high-pass filter of 0.5 Hz. ECG data were analyzed in Kubios HRV version 2.2 software (41). Data were manually controlled for artifacts and beat-to-beat (R-R) intervals were calculated with Kubios' automated QRS detection.

We calculated the RMSSD as a time domain measure, LF power (0.04–0.15 Hz) and HF power (0.15–0.4 Hz) as frequency-domain measures of HRV using fast Fourier transformation and are expressed in ms^2^. In addition, we also conducted a Poincaré plot analysis (plotting the RR_n_ interval values against RR_n+1_), and report the standard deviation (SD)1 and SD2 to quantify the shape of the plot. While SD2 describes the length of the long axis of the elliptical distribution and is assumed to reflect intermediate-term variability due to parasympathetic and sympathetic activity, SD1 describes the length of the transverse axis and is associated with short-term variability. Poincaré plot analysis was conducted for confirmatory purposes and results presented as Supplementary Material.

### Statistics

Data analyses were carried out using SPSS 22.0 statistical software (IBM Inc., Chicago, IL, USA). Since HRV values were non-normally distributed (Kolmogorov—Smirnov *p* < 0.05), data were log-transformed using a natural logarithm. Because we expected basal group differences, we initially calculated a univariate ANOVA 2 × 2 (“MDD × ACE”), with baseline HRV measurements averaged over both sessions as dependent variable. To adjust for those baseline differences and to have a measure of intervention-induced reactivity, we calculated change scores. For this purpose, we subtracted each participant's baseline value from the three respective post-intervention values. These derived change scores were analyzed with a mixed-measures analysis of variance (ANOVA) to investigate the effects of yohimbine on HRV reactivity, with the between-subjects factors “MDD” (MDD+ vs. MDD−) and “ACE” (ACE+ vs. ACE−) and the within-subjects factors “drug” (yohimbine vs. placebo) and “time” (+50, +90, and +120 min). Bonferroni corrected *post-hoc* tests were used to further analyze significant interaction effects. For testing of statistical significance, *p*-values smaller than 0.05 were considered to indicate significance. In case of violations of sphericity, reported *p*-values were Greenhouse-Geisser corrected.

## Results

### Demographic Characteristics

Groups did not differ with regard to sex, age, educational status, and the intake of hormonal contraception (see Table 1). Patients with MDD had overall significantly lower MADRS and BDI scores, compared to the group without MDD and irrespective of ACE. Participants with ACE had overall lower ETI scores, compared to the group without ACE and irrespective of MDD. Also, participants with ACE had overall lower CTQ scores, however, this measure was qualified by MDD status: participants with ACE and MDD had lower scores than those with ACE and no MDD [for a detailed description of the sample, see Kuehl et al. (42)].

**Table 1:** Sample characteristics of MDD patients with and without ACE and healthy participants with and without ACE.

	**MDD+/ACE+**	**MDD+/ACE−**	**MDD−/ACE+**	**MDD−/ACE−**	**Statistics**
	***N*** **= 25**	***N*** **= 24**	***N*** **= 27**	***N*** **= 48**	
Sex (f/m)	13/12	13/11	15/12	26/22	*p* = 0.99
Age (years; SD)	40.5 (11.2)	34.8 (10.9)	33.9 (10.6)	35.5 (10.3)	*p* = 0.12
Education (years; SD)	11.2 (1.5)	12.0 (1.4)	11.7 (1.5)	11.6 (1.6)	*p* = 0.29
Use of hormonal contraceptives (women, y/n)	4/9	3/10	5/10	5/21	*p* = 0.72

*MDD, Major depressive disorder; ACE, Adverse childhood experiences*.

### Heart Rate Variability

#### Basal Differences Between Groups

**Figure 1:**
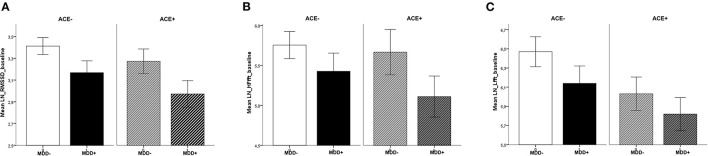
Baseline values across sessions, separate for MDD and ACE. For **(A)** RMSSD, **(B)** HF-HRV, and **(C)** LF-HRV (means ± 1SEM): Patients with MDD had lower basal RMSSD (compared to participants without MDD) and participants with ACE had lower basal LF-HRV (compared to participants without ACE).

Analysis of baseline values before the intervention revealed differences between the groups that were specific to the parameter studied. Compared to participants without MDD, patients with MDD had lower HRV values for RMSSD [*F*_(1,119)_ = 6.65, *p* = 0.01, $\eta_{\text{p}}^{2}$ = 0.06]. The effect for ACE, i.e., participants with vs. without ACE [*F*_(1,119)_ = 2.55, *p* = 0.11, $\eta_{\text{p}}^{2}$ = 0.02] and the interaction between MDD and ACE [*F*_(1,119)_ = 0.07, *p* = 0.79, $\eta_{\text{p}}^{2}$ < 0.01] were not significant. In the HF frequency band, there were no significant differences (main effects and interaction) between the groups, yet the effect for MDD was slightly above the significance threshold [MDD: *F*_(1,119)_ = 3.59, *p* = 0.06, $\eta_{\text{p}}^{2}$ = 0.03, ACE: *F*_(1,119)_ = 0.76, *p* = 0.39, $\eta_{\text{p}}^{2}$ = 0.01, MDD × ACE: *F*_(1,119)_ = 0.29, *p* = 0.63, $\eta_{\text{p}}^{2}$ < 0.01]. In the LF frequency band, HRV was lower in participants with ACE compared to those without ACE [*F*_(1,119)_ = 4.42, *p* = 0.04, $\eta_{\text{p}}^{2}$ = 0.04]. The effect for MDD [*F*_(1,119)_ = 2.35, *p* = 0.14, $\eta_{\text{p}}^{2}$ = 0.02] and the interaction between MDD and ACE [*F*_(1,119)_ = 0.11, *p* = 0.74, $\eta_{\text{p}}^{2}$ < 0.01] were not significant (see Figure 1).

#### Reactivity to Yohimbine

**Figure 2:**
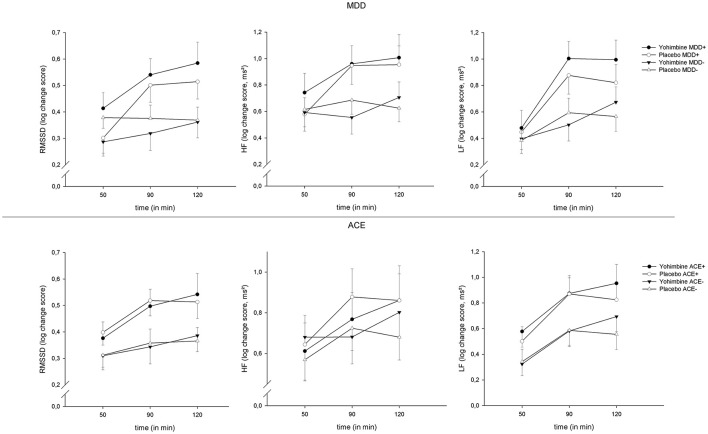
HRV reactivity 50, 90, and 120 min after yohimbine administration, separate for MDD (top) and ACE (bottom), and RMSSD (left), HF-HRV (center), and LF-HRV (right), means ± 1SEM.

Across all parameters, we found an increase in HRV over the factor “time” [RMSSD: *F*_(2,224)_ = 15.61, *p* < 0.001, $\eta_{\text{p}}^{2}$ = 0.12, HF: *F*_(2,224)_ = 8.59, *p* < 0.001, $\eta_{\text{p}}^{2}$ = 0.07, LF: *F*_(2,224)_ = 24.58, *p* < 0.001, $\eta_{\text{p}}^{2}$ = 0.18]; this effect was moderated by the between-subjects factor “time × MDD” [RMSSD: *F*_(2,224)_ = 8.46, *p* < 0.001, $\eta_{\text{p}}^{2}$ = 0.07, HF: *F*_(2,224)_ = 5.67, *p* < 0.01, $\eta_{\text{p}}^{2}$ = 0.05, LF: *F*_(2,224)_ = 4.27, *p* = 0.02, $\eta_{\text{p}}^{2}$ = 0.04]. To elucidate these interaction effects, *t*-tests were calculated to compare MDD+ vs. MDD− at each time interval: patients with MDD had a more pronounced increase in RMSSD at +90 min (*t*_(117)_ = 2.62, *p* = 0.01) and +120 min (*t*_(115)_ = 2.58, *p* = 0.01) and LF at +90 min (*t*_(117)_ = 2.79, *p* < 0.01). However, this change over time was independent of our experimental intervention and was found across both levels of the factor “drug” (see Figure 2). There were no significant main effects or interactions involving the factor “drug” (all *p* > 0.05).

## Discussion

In this study, we investigated the influence of a single administration of the alpha2-antagonist yohimbine on the reactivity of HRV in a sample with/without MDD and with/without ACE. A baseline HRV measurement under rest was obtained before the experimental intervention, as well as 3 measurements after the intervention at defined time intervals. As expected and in line with our hypotheses, basal group differences were found: patients with MDD had lower values in the parameters HF-HRV (trend level significance) and RMSSD, both measures that primarily reflect parasympathetic vagal activity. We further found lower basal values for the parameter LF-HRV in subjects with ACE. LF-HRV represents a joint influence of sympathetic and parasympathetic nervous system. There were no statistically significant interactions between MDD and ACE. The main objective of the study was the influence of yohimbine in these groups; therefore the change over time was measured and compared to baseline. In contrast to our hypotheses, we found no effect of yohimbine on HRV, neither as a main effect nor in interaction with MDD or ACE, for any parameter.

The lower basal HRV in depressed patients is well in line with previous research and complements the literature (5). The finding is indicative of persistent autonomic dysregulation in MDD. That we found effects in indicators of parasympathetic activity suggests that this impaired HRV is due to reduced vagal activity, which is also in line with existing research (43). However, we had also expected higher sympathetic activation in the group of MDD patients (44), which would have been expressed in lower LF-HRV. Contrary to our expectations, there was no effect of MDD for this parameter. We can only speculate about the reasons for this, but would like to point out that previous large meta-analyses came to similarly discrepant findings: while Kemp et al. (45) found reduced HF-HRV and no evidence for LF-HRV deviations in MDD, Brown et al. (30) analyzed studies specifically in older adults and, in contrast, found lower LF-HRV in MDD, with no difference in HF-HRV. However, a very recent meta-analysis indicated a significant reductions in both HF- and LF-HRV (5). Of note, there has been controversy about whether and to what extent antidepressants contribute to the reduction in HRV (46, 47). Since the patients in our study were all unmedicated, it can be concluded that HRV is decreased independently of this factor.

Interestingly, we found an inverse pattern for ACE: significantly lower basal values in the parameter LF-HRV–more strongly associated with sympathetic activation–and no differences in RMSSD and HF-HRV. Likewise, a recent meta-analysis could demonstrate reduced HRV in individuals with ACE, however, because the focus of this work was on dysregulations in resting-state vagal activity, only the vagally mediated parameters HF-HRV and RMSSD were included as outcome measures in the analysis (24). Yet, another recent paper also described a significant relationship between ACE and LF-HRV (48). Using path analysis models, the authors found a mediation of the relationship between CTQ scores (as a measure of ACE) and LF-HRV *via* affective lability and beta EEG activity, related to alertness and anxiety.

We found a significant increase in HRV over the duration of the experiment (factor “time”), which was more pronounced for patients with MDD (who again had lower basal values). However, this change was independent of our experimental manipulation (“drug”) and probably due to the situation in the laboratory and the orthostatic posture (sitting in the chair). In contrast to our hypothesis and to existing research, we did not find effects of yohimbine on HRV. Yeragani et al. (49) reported an increase in midfrequency (MF−) HRV (0.07–0.15 Hz, which basically corresponds to LF-HRV by contemporary standards) after a single dose administration of yohimbine. However, that study found the effect in patients with panic disorder, whose response to yohimbine was supposedly more pronounced when compared to our sample. In a further study, single dose administration of yohimbine increased LF-HRV (50). However, that study had a distinctly different research subject and procedure: it included only healthy participants and investigated the effects of meal-stimulated glucagon-like peptide-1 release on autonomic nervous functions, alone and in combination with yohimbine. Yohimbine was administered as a bolus and HRV was assessed in a postprandial period after overnight fasting and subsequent oral nutrient infusion. This null effect of our treatment was unexpected in that yohimbine induces a state similar to the immediate stress response through increased release of catecholamines and activation of the sympathetic nervous system, and since effects of stress on HRV are well-known from the literature (51).

In answering the question why, contrary to the hypotheses, no effect of yohimbine was found, several explanations can be thought of. The effect of yohimbine on the availability of noradrenaline corresponds to a part of the initial stress response, which is characterized by vagal withdrawal and increased sympathetic activation. However, this shift in ANS activity is accompanied by numerous other changes as part of the stress response. In particular, complex interactions with the hormones of the hypothalamic-pituitary-adrenal axis are evident (52), and isolated sympathetic activation may not be sufficient to map the effects of stress on HRV. We administered 10 mg of oral yohimbine, with dosages of 5–20 mg reported to affect cognitive processes in previous studies (53–56). However, this dosage may not have been sufficient to show effects on HRV; Bharucha et al. (50) found an effect on LF-HRV after an intravenous administration of 0.125 mg/kg over 10 min. Because of the specific effects of yohimbine on the sympathetic nervous system, we took LF-HRV as the dependent measure. This measure was originally used as a measure of sympathetic activation; however, this interpretation has become the subject of widespread controversy (57). While there is general consensus that high frequency (HF)-HRV is controlled by vagal activity and thus the parasympathetic nervous system, the neurophysiological, autonomic drivers influencing LF-HRV have been subject of recent debate (7). It can be considered certain that both branches of the ANS, sympathetic as well as parasympathetic, are reflected in LF-HRV (8). The relative proportion again depends on the recording conditions. The proportion of sympathetic influence is probably more pronounced during longer recordings. At short-term recordings such as the 5 min intervals in our study, LF-HRV is markedly influenced by feedback loops in the baroreflex system (7, 10). Potentially, an orthostatic challenge such as a standing posture as in Yeragani et al. (49) might have yielded different results. Again, while Yeragani et al. (49) reported their effect in patients with panic disorder, those were a population we specifically excluded. Future research could investigate these effects with higher doses of yohimbine, examining HRV over longer periods of time and under non-rest conditions.

Every single parameter of HRV measurement comes with its limitations. Problems with the interpretation of LF-HRV as a measure of sympathetic activity have already been discussed above. Normalization of LF-HRV and the use of normalized units (nu) have been proposed in the literature (58, 59). These are obtained by dividing LF power by the total power minus the (very low frequency) VLF power. While some authors proposed a closer conceptual correspondence between normalized LF-HRV and sympathetic activity, these claims have also been challenged. Data are distorted due to these mathematical transformation and the underlying assumption of autonomic reciprocity as well as the association between nu and LF (in absolute units) remain controversial (57). Furthermore, many studies of relevance to our research question have relied on (non-normalized) LF-HRV (5). Both RMSSD and HF-HRV reflect parasympathetic-vagal activation according to current research and are generally highly correlated. However, both have their specific advantages and disadvantages: compared to HF-HRV, RMSSD is less affected by respiratory influences (i.e., changes in breathing frequencies) and more robust to disturbances of the cardiac rhythm by ectopic beats, yet it is more dependent on basal heart period (42, 60–62). For these reasons, and for comparability to previous research findings of relevance to our topic, we included RMSSD, HF-HRV, and LF-HRV.

In conclusion, independent of MDD and ACE and potentially for the reasons mentioned above, no effect of yohimbine on HRV could be shown. However, we were able to demonstrate basal differences in HRV between the psychiatric groups. These form an interesting pattern, with low values in the parameters HF-HRV and RMSSD in MDD and lower LF-HRV in ACE. From this pattern, one could assume–relatively speaking–a stronger mediation by vagal dysregulation in MDD and influences of the sympathetic nervous system in ACE. However, this interpretation should be viewed with caution, in light of partial contrary findings in the literature as well as the unclear mechanisms underlying LF-HRV.

## Data Availability Statement

The raw data supporting the conclusions of this article will be made available by the authors, without undue reservation.

## Ethics Statement

The studies involving human participants were reviewed and approved by German Psychological Society (Deutsche Gesellschaft für Psychologie). The patients/participants provided their written informed consent to participate in this study.

## Author Contributions

CD, CO, KW, and LK conceptualized, planned, and designed the study. CD and LK conducted the study and analyzed the data. CD wrote the manuscript.

## Funding

LK, KW, and CO were supported by the grant effects of increased noradrenergic activity by yohimbine administration on learning and attention in patients with major depression disorder, funded by the German Research Foundation (Deutsche Forschungsgemeinschaft/DFG), project KU 3106/2-1.

## Conflict of Interest

The authors declare that the research was conducted in the absence of any commercial or financial relationships that could be construed as a potential conflict of interest.

## Publisher's Note

All claims expressed in this article are solely those of the authors and do not necessarily represent those of their affiliated organizations, or those of the publisher, the editors and the reviewers. Any product that may be evaluated in this article, or claim that may be made by its manufacturer, is not guaranteed or endorsed by the publisher.

## References

[1] Brush CJ Olson RL Ehmann PJ Bocchine AJ Bates ME Buckman JF. Lower resting cardiac autonomic balance in young adults with current major depression. Psychophysiology. (2019) 56:e13385. 10.1111/psyp.13385 31020679 PMC6650364

[2] Koschke M Boettger MK Schulz S Berger S Terhaar J Voss A. Autonomy of autonomic dysfunction in major depression. Psychosom Med. (2009) 71:852–60. 10.1097/PSY.0b013e3181b8bb7a 19779146

[3] Sgoifo A Carnevali L Alfonso Mde L Amore M. Autonomic dysfunction and heart rate variability in depression. Stress. (2015) 18:343–52. 10.3109/10253890.2015.1045868 26004818

[4] Gold SM Köhler-Forsberg O Moss-Morris R Mehnert A Miranda JJ Bullinger M. Comorbid depression in medical diseases. Nat Rev Dis Primers. (2020) 6:69. 10.1038/s41572-020-0200-2 32820163

[5] Koch C Wilhelm M Salzmann S Rief W Euteneuer F. A meta-analysis of heart rate variability in major depression. Psychol Med. (2019) 49:1948–57. 10.1017/S0033291719001351 31239003

[6] Schiweck C Piette D Berckmans D Claes S Vrieze E. Heart rate and high frequency heart rate variability during stress as biomarker for clinical depression. A systematic review. Psychol Med. (2019) 49:200–11. 10.1017/S0033291718001988 30134999

[7] Shaffer F McCraty R Zerr CL. A healthy heart is not a metronome: an integrative review of the heart's anatomy and heart rate variability. Front Psychol. (2014) 5:1040. 10.3389/fpsyg.2014.01040 25324790 PMC4179748

[8] Laborde S Mosley E Thayer JF. Heart rate variability and cardiac vagal tone in psychophysiological research - recommendations for experiment planning, data analysis, and data reporting. Front Psychol. (2017) 8:213. 10.3389/fpsyg.2017.00213 28265249 PMC5316555

[9] Quintana DS Heathers JA. Considerations in the assessment of heart rate variability in biobehavioral research. Front Psychol. (2014) 5:805. 10.3389/fpsyg.2014.00805 25101047 PMC4106423

[10] Shaffer F Ginsberg JP. An overview of heart rate variability metrics and norms. Front Public Health. (2017) 5:258. 10.3389/fpubh.2017.00258 29034226 PMC5624990

[11] Holland N Robbins TW Rowe JB. The role of noradrenaline in cognition and cognitive disorders. Brain. (2021) 144:2243–56. 10.1093/brain/awab111 33725122 PMC8418349

[12] Cottingham C Wang Q. alpha2 adrenergic receptor dysregulation in depressive disorders: implications for the neurobiology of depression and antidepressant therapy. Neurosci Biobehav Rev. (2012) 36:2214–25. 10.1016/j.neubiorev.2012.07.011 22910678 PMC3508310

[13] Otte C Gold SM Penninx BW Pariante CM Etkin A Fava M. Major depressive disorder. Nat Rev Dis Primers. (2016) 2:16065. 10.1038/nrdp.2016.65 27629598

[14] Goddard AW Ball SG Martinez J Robinson MJ Yang CR Russell JM. Current perspectives of the roles of the central norepinephrine system in anxiety and depression. Depress Anxiety. (2010) 27:339–50. 10.1002/da.20642 19960531

[15] Nemeroff CB. Recent advances in the neurobiology of depression. Psychopharmacol Bull. (2002) 36 (Suppl 2):6–23. 12490820

[16] Seki K Yoshida S Jaiswal MK. Molecular mechanism of noradrenaline during the stress-induced major depressive disorder. Neural Regen Res. (2018) 13:1159–69. 10.4103/1673-5374.235019 30028316 PMC6065220

[17] Ordway GA Schenk J Stockmeier CA May W Klimek V. Elevated agonist binding to alpha2-adrenoceptors in the locus coeruleus in major depression. Biol Psychiatry. (2003) 53:315–23. 10.1016/S0006-3223(02)01728-6 12586450

[18] Rivero G Gabilondo AM Garcia-Sevilla JA La Harpe R Callado LF Meana JJ. Increased alpha2- and beta1-adrenoceptor densities in postmortem brain of subjects with depression: differential effect of antidepressant treatment. J Affect Disord. (2014) 167:343–50. 10.1016/j.jad.2014.06.016 25020269

[19] Saboory E Ghasemi M Mehranfard N. Norepinephrine, neurodevelopment and behavior. Neurochem Int. (2020) 135:104706. 10.1016/j.neuint.2020.104706 32092327

[20] Kuras YI McInnis CM Thoma MV Chen X Hanlin L Gianferante D. Increased alpha-amylase response to an acute psychosocial stress challenge in healthy adults with childhood adversity. Dev Psychobiol. (2017) 59:91–8. 10.1002/dev.21470 27577885 PMC5651411

[21] Otte C Neylan TC Pole N Metzler T Best S Henn-Haase C. Association between childhood trauma and catecholamine response to psychological stress in police academy recruits. Biol Psychiatry. (2005) 57:27–32. 10.1016/j.biopsych.2004.10.009 15607297

[22] Lee RJ Fanning JR Coccaro EF. GH response to intravenous clonidine challenge correlates with history of childhood trauma in personality disorder. J Psychiatr Res. (2016) 76:38–43. 10.1016/j.jpsychires.2015.11.009 26874268

[23] Silva RC Maffioletti E Gennarelli M Baune BT Minelli A. Biological correlates of early life stressful events in major depressive disorder. Psychoneuroendocrinology. (2020) 125:105103. 10.1016/j.psyneuen.2020.105103 33360031

[24] Sigrist C Mürner-Lavanchy I Peschel SKV Schmidt SJ Kaess M Koenig J. Early life maltreatment and resting-state heart rate variability: a systematic review and meta-analysis. Neurosci Biobehav Rev. (2021) 120:307–34. 10.1016/j.neubiorev.2020.10.026 33171141

[25] Tell D Mathews HL Burr RL Witek Janusek L. During stress, heart rate variability moderates the impact of childhood adversity in women with breast cancer. Stress. (2018) 21:179–87. 10.1080/10253890.2018.1424132 29385886

[26] Voellmin A Winzeler K Hug E Wilhelm FH Schaefer V Gaab J. Blunted endocrine and cardiovascular reactivity in young healthy women reporting a history of childhood adversity. Psychoneuroendocrinology. (2015) 51:58–67. 10.1016/j.psyneuen.2014.09.008 25290347

[27] Stone LB Amole MC Cyranowski JM Swartz HA. History of childhood emotional abuse predicts lower resting-state high-frequency heart rate variability in depressed women. Psychiatry Res. (2018) 269:681–7. 10.1016/j.psychres.2018.08.106 30273892 PMC6223021

[28] Cyranowski JM Hofkens TL Swartz HA Salomon K Gianaros PJ. Cardiac vagal control in nonmedicated depressed women and nondepressed controls: impact of depression status, lifetime trauma history, and respiratory factors. Psychosom Med. (2011) 73:336–43. 10.1097/PSY.0b013e318213925d 21364194 PMC3090496

[29] Winzeler K Voellmin A Hug E Kirmse U Helmig S Princip M. Adverse childhood experiences and autonomic regulation in response to acute stress: the role of the sympathetic and parasympathetic nervous systems. Anxiety Stress Coping. (2017) 30:145–54. 10.1080/10615806.2016.1238076 27653030

[30] Brown L Karmakar C Gray R Jindal R Lim T Bryant C. Heart rate variability alterations in late life depression: a meta-analysis. J Affect Disord. (2018) 235:456–66. 10.1016/j.jad.2018.04.071 29679898

[31] Wittchen H-U Zaudig M Fydrich T. SKID. Strukturiertes Klinisches Interview für DSM-IV. Achse I und II. Handanweisung. Göttingen: Hogrefe (1997). 10.1026//0084-5345.28.1.68

[32] Montgomery SA Asberg M. A new depression scale designed to be sensitive to change. Br J Psychiatry. (1979) 134:382–9. 10.1192/bjp.134.4.382 444788

[33] Williams JB Kobak KA. Development and reliability of a structured interview guide for the Montgomery Asberg Depression Rating Scale (SIGMA). Br J Psychiatry. (2008) 192:52–8. 10.1192/bjp.bp.106.032532 18174510

[34] Beck AT Steer RA Hautzinger M. Beck-Depressions-Inventar. testhandbuch: Huber (1994).

[35] Bremner JD Vermetten E Mazure CM. Development and preliminary psychometric properties of an instrument for the measurement of childhood trauma: the Early Trauma Inventory. Depress Anxiety. (2000) 12:1–12. 10.1002/1520-6394(2000)12:1<1::AID-DA1>3.0.CO;2-W 10999240

[36] Wingenfeld K Schafer I Terfehr K Grabski H Driessen M Grabe H. [The reliable, valid and economic assessment of early traumatization: first psychometric characteristics of the German version of the Adverse Childhood Experiences Questionnaire (ACE)]. Psychother Psychosom Med Psychol. (2011) 61:e10–14. 10.1055/s-0030-1263161 20878600

[37] Bernstein DP Stein JA Newcomb MD Walker E Pogge D Ahluvalia T. Development and validation of a brief screening version of the Childhood Trauma Questionnaire. Child Abuse Negl. (2003) 27:169–90. 10.1016/S0145-2134(02)00541-0 12615092

[38] Wingenfeld K Spitzer C Mensebach C Grabe HJ Hill A Gast U. The German version of the Childhood Trauma Questionnaire (CTQ): preliminary psychometric properties. Psychother Psychosom Med Psychol. (2010) 60:442–50. 10.1055/s-0030-1247564 20200804

[39] Deuter CE Nowacki J Wingenfeld K Kuehl LK Finke JB Dziobek I. The role of physiological arousal for self-reported emotional empathy. Auton Neurosci. (2018) 214:9–14. 10.1016/j.autneu.2018.07.002 30104144

[40] Nowacki J Duesenberg M Deuter CE Otte C Wingenfeld K. Delayed effects of psychosocial stress on risk taking. Stress. (2019) 22:446–54. 10.1080/10253890.2019.1593364 30961412

[41] Tarvainen MP Niskanen JP Lipponen JA Ranta-Aho PO Karjalainen PA. Kubios HRV–heart rate variability analysis software. Comput Methods Programs Biomed. (2014) 113:210–20. 10.1016/j.cmpb.2013.07.024 24054542

[42] Kuehl LK Deuter CE Richter S Schulz A Ruddel H Schachinger H. Two separable mechanisms are responsible for mental stress effects on high frequency heart rate variability: an intra-individual approach in a healthy and a diabetic sample. Int J Psychophysiol. (2015) 95:299–303. 10.1016/j.ijpsycho.2014.12.003 25500224

[43] Stapelberg NJ Hamilton-Craig I Neumann DL Shum DH McConnell H. Mind and heart: heart rate variability in major depressive disorder and coronary heart disease - a review and recommendations. Aust N Z J Psychiatry. (2012) 46:946–57. 10.1177/0004867412444624 22528974

[44] Lehofer M Moser M Hoehn-Saric R McLeod D Liebmann P Drnovsek B. Major depression and cardiac autonomic control. Biol Psychiatry. (1997) 42:914–9. 10.1016/S0006-3223(96)00494-5 9359977

[45] Kemp AH Quintana DS Gray MA Felmingham KL Brown K Gatt JM. Impact of depression and antidepressant treatment on heart rate variability: a review and meta-analysis. Biol Psychiatry. (2010) 67:1067–74. 10.1016/j.biopsych.2009.12.012 20138254

[46] Huang WL Liao SC Kuo TB Chang LR Chen TT Chen IM. The effects of antidepressants and quetiapine on heart rate variability. Pharmacopsychiatry. (2016) 49:191–8. 10.1055/s-0042-102964 27023265

[47] Kemp AH Fráguas R Brunoni AR Bittencourt MS Nunes MA Dantas EM. Differential associations of specific selective serotonin reuptake inhibitors with resting-state heart rate and heart rate variability: implications for health and well-being. Psychosom Med. (2016) 78:810–8. 10.1097/PSY.0000000000000336 27219492

[48] Jin MJ Kim JS Kim S Hyun MH Lee SH. An integrated model of emotional problems, beta power of electroencephalography, and low frequency of heart rate variability after childhood trauma in a non-clinical sample: a path analysis study. Front Psychiatry. (2017) 8:314. 10.3389/fpsyt.2017.00314 29403401 PMC5786859

[49] Yeragani VK Berger R Pohl R Srinivasan K Balon R Ramesh C. Effects of yohimbine on heart rate variability in panic disorder patients and normal controls: a study of power spectral analysis of heart rate. J Cardiovasc Pharmacol. (1992) 20:609–18. 10.1097/00005344-199210000-00015 1280718

[50] Bharucha AE Charkoudian N Andrews CN Camilleri M Sletten D Zinsmeister AR. Effects of glucagon-like peptide-1, yohimbine, and nitrergic modulation on sympathetic and parasympathetic activity in humans. Am J Physiol Regul Integr Comp Physiol. (2008) 295:R874–880. 10.1152/ajpregu.00153.2008 18596108 PMC2536850

[51] Kim HG Cheon EJ Bai DS Lee YH Koo BH. Stress and heart rate variability: a meta-analysis and review of the literature. Psychiatry Investig. (2018) 15:235–45. 10.30773/pi.2017.08.17 29486547 PMC5900369

[52] Chen FR Raine A Granger DA. The within-person coordination of HPA and ANS activity in stress response: Relation with behavior problems. Psychoneuroendocrinology. (2020) 121:104805. 10.1016/j.psyneuen.2020.104805 32745923

[53] O'Carroll RE Drysdale E Cahill L Shajahan P Ebmeier KP. Stimulation of the noradrenergic system enhances and blockade reduces memory for emotional material in man. Psychol Med. (1999) 29:1083–8. 10.1017/S0033291799008703 10576300

[54] Soeter M Kindt M. Noradrenergic enhancement of associative fear memory in humans. Neurobiol Learn Mem. (2011) 96:263–71. 10.1016/j.nlm.2011.05.003 21624479

[55] Wingenfeld K Kuffel A Uhlmann C Terfehr K Schreiner J Kuehl LK. Effects of noradrenergic stimulation on memory in patients with major depressive disorder. Stress. (2013) 16:191–201. 10.3109/10253890.2012.708951 22746337

[56] Soeter M Kindt M. Stimulation of the noradrenergic system during memory formation impairs extinction learning but not the disruption of reconsolidation. Neuropsychopharmacology. (2012) 37:1204–15. 10.1038/npp.2011.307 22169947 PMC3306881

[57] Reyes del Paso GA Langewitz W Mulder LJ van Roon A Duschek S. The utility of low frequency heart rate variability as an index of sympathetic cardiac tone: a review with emphasis on a reanalysis of previous studies. Psychophysiology. (2013) 50:477–87. 10.1111/psyp.12027 23445494

[58] Malliani A Pagani M Lombardi F. Importance of appropriate spectral methodology to assess heart rate variability in the frequency domain. Hypertension. (1994) 24:140–2. 10.1161/01.HYP.24.1.140 8021002

[59] Montano N Porta A Cogliati C Costantino G Tobaldini E Casali KR. Heart rate variability explored in the frequency domain: a tool to investigate the link between heart and behavior. Neurosci Biobehav Rev. (2009) 33:71–80. 10.1016/j.neubiorev.2008.07.006 18706440

[60] Berntson GG Lozano DL Chen YJ. Filter properties of root mean square successive difference (RMSSD) for heart rate. Psychophysiology. (2005) 42:246–52. 10.1111/j.1469-8986.2005.00277.x 15787862

[61] Hill LK Siebenbrock A. Are all measures created equal? Heart rate variability and respiration - biomed 2009. Biomed Sci Instrum. (2009) 45:71–6. 19369742

[62] Penttilä J Helminen A Jartti T Kuusela T Huikuri HV Tulppo MP. Time domain, geometrical and frequency domain analysis of cardiac vagal outflow: effects of various respiratory patterns. Clin Physiol. (2001) 21:365–76. 10.1046/j.1365-2281.2001.00337.x 11380537

