# Supplementary material for: Yohimbine-Induced Reactivity of Heart Rate Variability in Unmedicated Depressed Patients With and Without Adverse Childhood Experience
Source: Front Psychiatry. 2021 Dec 16;12:734904. doi: 10.3389/fpsyt.2021.734904 (PMC8717379; doi:10.3389/fpsyt.2021.734904)
Supplement: Supplementary file 1 [file Data_Sheet_1.PDF]

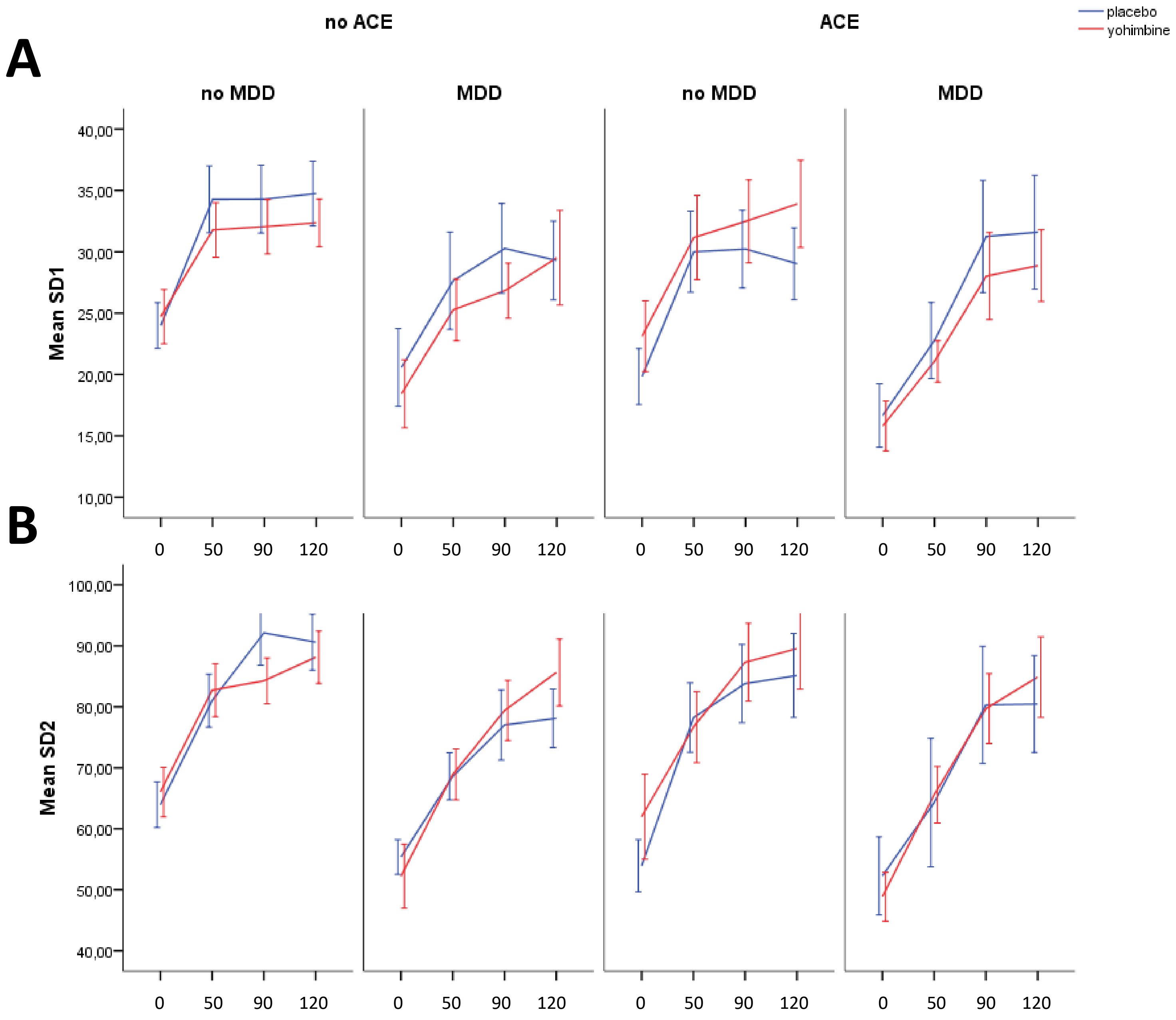

Poincaré plot analysis with SD1 in the upper row (A) and SD2 in the lower row (B) for the four measuring times: 0 = baseline. 50. 90 and 120 min. +/- 1SE

SD1 - *Tests of Within-Subjects Effects*

	df	F	*p*	η_p^2
yoh	1	.244	.622	.002
yoh * ACE	1	2.327	.130	.020
yoh * MDD	1	1.510	.222	.013
yoh * ACE * MDD	1	.939	.335	.008
Error(yoh)	112			
time	3	67.897	.000	.377
time * ACE	3	1.099	.337	.010
time * MDD	3	2.702	.067	.024
time * ACE * MDD	3	.720	.492	.006
Error(time)	336			
yoh * time	3	1.023	.363	.009
yoh * time * ACE	3	.096	.915	.001
yoh * time * MDD	3	.220	.811	.002
yoh * time * ACE * MDD	3	1.324	.268	.012
Error(yoh*time)	336			

SD1 - *Tests of Between-Subjects Effects*

	df	F	*p*	η_p^2
Intercept	1	438.949	.000	.797
ACE	1	.860	.356	.008
MDD	1	4.414	.038	.038
ACE * MDD	1	.045	.832	.000
Error	112			

SD2 - *Tests of Within-Subjects Effects*

	df	F	*p*	η_p^2
yoh	1	.294	.589	.003
yoh * ACE	1	.600	.440	.005
yoh * MDD	1	.017	.898	.000
yoh * ACE * MDD	1	.686	.409	.006
Error(yoh)	112			
time	3	100.480	.000	.473
time * ACE	3	.423	.715	.004
time * MDD	3	.977	.397	.009
time * ACE * MDD	3	.069	.967	.001
Error(time)	336			
yoh * time	3	.448	.704	.004
yoh * time * ACE	3	.344	.778	.003
yoh * time * MDD	3	1.388	.248	.012
yoh * time * ACE * MDD	3	.699	.543	.006
Error(yoh*time)	336			

SD2 - *Tests of Between-Subjects Effects*

	df	F	*p*	η_p^2
Intercept	1	1019.607	.000	.901
ACE	1	.642	.425	.006
MDD	1	4.870	.029	.042
ACE * MDD	1	.001	.975	.000
Error	112			
